# Pregnancy-Related Complications in Women with Recurrent Pregnancy Loss: A Prospective Cohort Study

**DOI:** 10.3390/jcm9092833

**Published:** 2020-09-01

**Authors:** Carlo Ticconi, Adalgisa Pietropolli, Monia Specchia, Elena Nicastri, Carlo Chiaramonte, Emilio Piccione, Giovanni Scambia, Nicoletta Di Simone

**Affiliations:** 1 Department of Surgical Sciences, Section of Gynecology and Obstetrics, University Tor Vergata, 00168 Rome, Italy; pietropolli@med.uniroma2.it (A.P.); elena.nicastri@gmail.com (E.N.); piccione@med.uniroma2.it (E.P.); 2 Unità Operativa Complessa (U.O.C.) di Ostetricia e Patologia Ostetrica, Dipartimento di Scienze della Salute della Donna, del Bambino e di Sanità Pubblica, Fondazione Policlinico Universitario A.Gemelli Istituto di Ricerca e Cura a Carattere Scientifico (IRCCS), 00168 Rome, Italy; monia.specchia@libero.it (M.S.); nicoletta.disimone@policlinicogemelli.it (N.D.S.); 3 Istituto di Clinica Ostetrica e Ginecologica, Università Cattolica del Sacro Cuore, 00168 Rome, Italy; giovanni.scambia@policlinicogemelli.it; 4 Department of Biomedicine and Prevention, University Tor Vergata, Viale Oxford 81, 00133 Rome, Italy; chiaramonte.carlo43@gmail.com; 5 U.O.C. di Ginecologia Oncologica, Dipartimento di Scienze della Salute della Donna, del Bambino e di Sanità Pubblica, Fondazione Policlinico Universitario A. Gemelli IRCCS, 00168 Rome, Italy

**Keywords:** recurrent pregnancy loss, pregnancy complications, obstetrics, reproductive health

## Abstract

The aim of this prospective cohort study was to determine whether women with recurrent pregnancy loss (RPL) have an increased risk of pregnancy complications compared to normal pregnant women. A total of 1092 singleton pregnancies were followed, 431 in women with RPL and 661 in normal healthy women. The prevalence of the following complications was observed: threatened miscarriage, miscarriage, cervical insufficiency, chromosomal/genetic abnormalities, fetal anomalies, oligohydramnios, polyhydramnios, fetal growth restriction, intrauterine fetal death, gestational diabetes mellitus (GDM), preeclampsia, placenta previa, abruptio placentae, pregnancy-related liver disorders, and preterm premature rupture of the membranes. The odds ratio and 95% CI for each pregnancy complication considered were determined by comparing women with RPL and normal healthy women. Women with RPL had an overall rate of pregnancy complications higher than normal women (OR = 4.37; 95% CI: 3.353–5.714; *p* < 0.0001). Their risk was increased for nearly all the conditions considered. They also had an increased risk of multiple concomitant pregnancy complications (OR = 4.64; 95% CI: 3.10–6.94, *p* < 0.0001). Considering only women with RPL, women with ≥3 losses had a higher risk of pregnancy complications than women with two losses (OR = 1.269; 95% CI: 1.112–2.386, *p* < 0.02). No differences were found in the overall risk of pregnancy complications according to the type, explained or unexplained, of RPL. Women with secondary RPL had an increased risk of GDM than women with primary RPL. Pregnancy in women with RPL should be considered at high risk.

## 1. Introduction

Recurrent pregnancy loss (RPL), defined as the spontaneous loss of two or more pregnancies (according to the American Society for Reproductive Medicine [1]) or the loss of two or more pregnancies before the 24th week of gestation (according to the European Society of Human Reproduction and Embryology [2]), presents several still incompletely defined aspects. Among these is the outcome of the successive pregnancy in women with a history of RPL. Indeed, there is considerable discrepancy between the reported birth rates and the rates of gestational complications of the successive pregnancy in women with RPL. The likelihood of a live birth in the successive pregnancy in untreated women with RPL has been reported to range 42–86% after three miscarriages and decreases with increasing the number of pregnancy losses, reaching only 23–51% after ≥5 losses [3]. This observation suggests that the number of miscarriages—a likely indicator of the gravity of the condition—is a major determinant of the reproductive success of women with RPL; in fact, it has been reported that the live birth rates in the successive pregnancy in women with two consecutive losses is around 75% [4,5]. On the other hand, several studies and reviews investigating the outcome of the successive pregnancy in women with RPL found that it was burdened by many obstetric and perinatal complications which occurred more frequently than in normal control women without RPL [6,7,8,9], even though this finding has not been observed in all studies [10]. Therefore, it is difficult for the clinicians to ensure a clear counseling, in terms of prognosis, to women with RPL about the subsequent pregnancy once the diagnostic workup has been completed. Whereas the outcome of the subsequent pregnancy in women with RPL in terms of live birth, labor, and perinatal complications is well established [3,6,8], less information is available on the obstetric risks that can occur in these women during their pregnancy before labor. Moreover, limited information is available concerning the gestational complications in women with RPL with regard to their specific features (primary/secondary or explained/unexplained RPL).

The present study was carried out to investigate the outcome of the first pregnancy in women with RPL after their referral compared with healthy pregnant women without RPL, with specific application to the gestational complications and to the particular characteristics of these women. Further information on this issue could allow a more comprehensive counseling of women with RPL and could help to better clarify whether these women actually need a more thorough monitoring throughout their pregnancy.

## 2. Methods

### 2.1. Subjects and Study Design

This prospective, observational, study has been carried out to investigate the occurrence rates of major gestational complications in a cohort of women with RPL compared to normal healthy women without RPL followed during their first subsequent pregnancy after referral. The study subjects were enrolled from 1 January 2017 to 31 January 2020.

Overall 1782 women were initially enrolled in the study. They attended as outpatients the Gynecology and Obstetrics Unit of the Policlinico Tor Vergata University Hospital or the Università Cattolica of the Sacred Hearth at the Policlinico Gemelli Hospital of Rome, Italy. In both Hospitals, the women with RPL were followed at the RPL Units, whereas the control women were followed throughout their pregnancy in the Low-Risk Obstetric Clinics. The study subjects were divided into two groups:

Group 1, RPL (*n* = 1030): non-pregnant women with RPL, enrolled at their first visit carried out to investigate the possible causes/risk factors of the RPL. The women of this group who entered in pregnancy were followed during their gestation. Group 2, Control (*n* = 752): pregnant normal women with low risk pregnancy who had at least one uncomplicated pregnancy at term with any previous pregnancy loss. They were followed throughout their gestation. All the control women have had at least two pregnancies at term without any losses and were selected consecutively.

**Figure 1:**
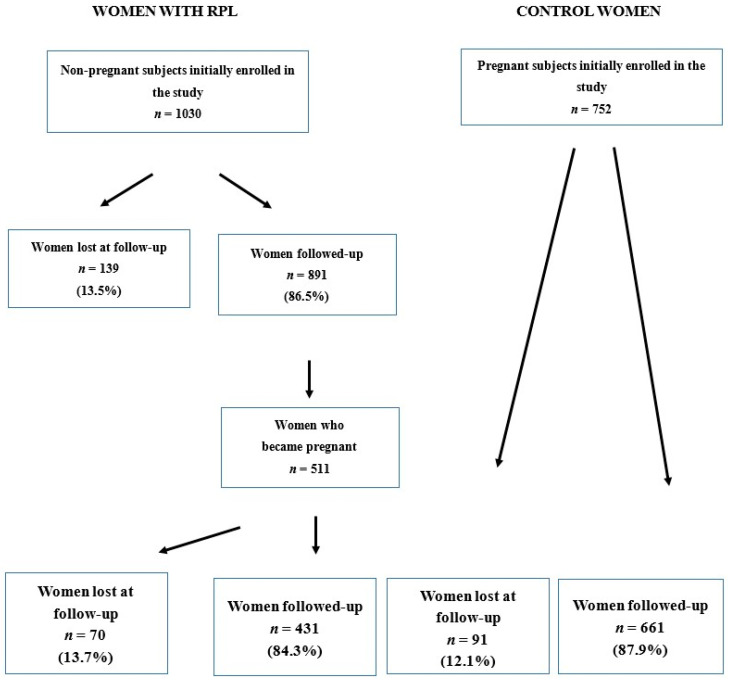
Distribution of the subjects enrolled in the study according to the follow-up.

Of the initial population of enrolled women, 139 women (13.5%) with RPL were lost at follow-up without any information on whether they get pregnant. Of the remaining 891 women followed-up, 511 (57.3%) become pregnant; of these women, 70 (13.7%) were lost at the successive follow-up while pregnant, whereas 431 (84.3%) were followed-up during their pregnancy. Ninety-one women (12.1%) of the control group were lost at follow-up during their pregnancy, while 661 women (87.9%) were followed up. Therefore, the final number of pregnant women included in the study was 1092. These numbers have been reported for clarity in Figure 1.

The final number of 1092 pregnant women to be included was calculated by taking into account the following conditions: (a) the null hypothesis (cases and controls have the same pregnancy complications) is refused if the difference between the means of the cases and of the controls (size effect) is ≥13.8% of the jointed variance of the two distributions; (b) the verification test is two-tailed Student’s *t*-test in which α = 0.05 and β = 0.10, with a power = 90%. With the above conditions, the overall number of women to be included (cases and controls) is no less than 1092.

The women with RPL were investigated according to a standardized diagnostic protocol already reported in detail [11,12] which included the collection of general and obstetrical history, gynecologic examination with a pelvic ultrasound scan, karyotype of both partners, hysteroscopy, hormonal profile, autoantibodies panel, metabolic evaluation, and screening for coagulation and thrombophilic disorders. The diagnostic workup was aimed to identify defined and probable causes of RPL. The women with RPL with treatable causes (medical and/or surgical) were treated according to the European Society of Human Reproduction and Embryology (ESHRE) Guidelines [2]. Treatments in women who were enrolled before the publication of these guidelines were changed accordingly.

The women in the control group were followed throughout the pregnancy until term in the low-risk pregnancy unit according to the standardized protocol used in our units, which complies with the National Institute for Health and Clinical Excellence (NICE) Clinical Guidelines [13].

The present study was carried out in accordance with the Declaration of Helsinki, modified Tokyo 2004, and was approved by the Institutional Review Board (IRB) of Policlinico Tor Vergata University Hospital (protocol number: 230/19). All women gave their informed written consent to the study.

### 2.2. Definitions and Inclusion/Exclusion Criteria—Data Collection and Handling

RPL was defined according to the ESHRE 2017 Guidelines [2]. RPL was defined as unexplained when no definite cause could be found at the end of the diagnostic workup. Primary RPL was defined as the absence of previous pregnancy at term or beyond the 24 weeks of gestation; secondary RPL was defined as the presence of two or more consecutive losses occurring in women with a previous child or whose previous pregnancy reached the 24 weeks of gestational age.

All the women of both groups with pre-existing diabetes and hypertension before the onset of pregnancy were excluded, in order to avoid confounding factors as much as possible, since the objective of the study was to investigate with specific attention the pregnancy-related complications in relation to RPL. Women with multiple pregnancies were also excluded from the study.

The following additional inclusion criteria were followed for control women: absence of any pre-existing medical conditions, no previous gynecologic surgery, no assumption of drugs before pregnancy. The definitions of the pregnancy complications of interest for the present study are reported in Table 1.

All the collected data of interest for the present study were reported in a preconceived template. A computerized database available for the successive analyses was then constructed. Any collected information was anonymized and de-identified prior to analysis.

### 2.3. Statistical Analysis

Data are presented as means ± standard deviation (SD) or percentages or proportions, or odds ratios (OR) and 95% confidence intervals (CI) as appropriate. Statistical analysis was carried out by using Student’s *t*-test and chi-square test. Bravais–Pearson coefficient was determined to analyze correlations. The software used was the Statistical Software SPSS release 23 (IBM^®^, Armonk, NY, USA). The effect of age and BMI for each complication is counted as percentages of the total population of women (cases and controls); the data were elaborated by using the method of analysis of the averages; by using this approach, the “effect size” expressed in percentages maintains the same dimensional magnitude of the original data. This statistical elaboration has been applied separately for the two above factors in relation to each complication; the results have been analyzed by Student’s under the usual hypotheses of normality and homogeneity of the corresponding distributions. Significance was set at *p* < 0.05.

## 3. Results

### 3.1. Clinical Characteristics of Study Women

Overall 1092 singleton pregnancies were followed (431 in women with RPL and 661 in normal healthy controls). The major clinical characteristics of the study women are reported in Table 2. Ethnicity distribution was not different between the two study groups (chi-square test = 4.41, *p* = 0.29, not significant difference). No difference was found between the rates of women with two (48.5%) or ≥3 (51.5%) losses in the population of women with RPL (chi-square test = 0.205, not significant). Conversely, significant differences were found in the rates of women with primary vs. secondary RPL (chi-square test = 28.72, *p* < 0.001), as well as in the rates of women with explained vs. unexplained RPL (chi-square test = 11.35, *p* < 0.001). No differences were found between the two groups in the rates of pregnant women lost at follow-up (Figure 1; chi-square test = 0.78, not significant). The mean length of follow-up was similar in the two groups (Table 2).

### 3.2. Pregnancy Complications in Women with RPL and in Control Women

No maternal deaths were observed in the overall study population. Live births were 371/431 (86%) in women with RPL and 643/661 (97.2%) in women of control group. Women with RPL during their subsequent pregnancy after referral, had a significantly increased risk of not having a live birth compared with control women (OR = 5.77, 95% CI: 3.359–9.933, *p* < 0.0001). Women with RPL also had a significantly higher overall rate of pregnancy complications (231/431, (53.6%) than control women (138/661, 20.9%): OR = 4.37, 95% CI: 3.353–5.714; *p* < 0.0001).

The rates by specific complications are reported in Table 3. The factorial analysis (reported in detail in supplemental Tables S1 and S2) has been carried out to ascertain the effect of age and BMI, that were higher in RPL than in control women; it revealed that both age and BMI had a significant effect on the distribution of nearly all of the above considered complications. This effect was particularly relevant in: (a) the case of age for spontaneous miscarriage; and (b) the case of BMI, for spontaneous miscarriage, chromosomal abnormalities, fetal growth restriction, gestational diabetes mellitus, and preeclampsia.

The fetal chromosomal abnormalities detected were: trisomy 21; trisomy 22; monosomy 45, X0; trisomy 47, XXY; autosomal triploidy. The fetal anomalies detected during prenatal ultrasonography were: clinodactyly, pre-axial polydactyly, hydrops fetalis, interventricular septal defect, tricuspidal insufficiency, micrognathia, trigonocephaly, femoral heterometry, liver calcifications, cystic hygroma, bilateral pyelectasis, alterations of head circumference, persistence of the right umbilical vein. The detail of the pregnancy-related liver disorders is reported as Supplementary material (Supplementary Table S3).

The effect of the number of previous losses on the risk for each specific pregnancy complication was also investigated and the results have been reported in Table 4. A “gravity-response” effect was clearly evident in the case of spontaneous miscarriage, cervical insufficiency, chromosomal abnormalities and preterm PROM.

The number of women who had more than one pregnancy complication was higher in the RPL group (93/431, 21.57%) than in the control group (37/661, 5.59%; OR = 4.64, 95% CI: 3.10–6.94, *p* < 0.0001). The detailed rates of concomitant pregnancy complications in study women are reported in Table 5.

### 3.3. Specific Features of Pregnancy Complications in Women with RPL

Two hundred and nine women with RPL had two previous pregnancy losses and 222 had three or more previous losses. When the women with RPL were stratified in two major groups according to the number of previous losses (two and ≥3) the pregnancy complication rate in the women with ≥3 losses (132/222, 59.45%) was higher than that of women with two losses (99/209, 47.36%; OR = 1.269; 95% CI: 1.112–2.386, *p* < 0.02). The detailed rates of pregnancy complications by the number of previous losses are shown in Supplementary Table S4.

The pregnancy complications in women with RPL were then stratified by the main diagnostic categories, i.e., explained and unexplained. One hundred and thirty-seven (52.89%) out of the 259 women who had an explained RPL and 94 (54.65%) out of the 172 women who had an unexplained RPL had a pregnancy complication, respectively. This overall difference was not significant (OR = 0.931; 95% CI: 0.632–1.371; *p* = 0.72). However, when the two populations of women with RPL were analyzed by specific type of complications, women with unexplained RPL had an increased risk to develop preeclampsia (OR = 2.35, 95% CI: 1.26–4.38, *p* < 0.05) and pregnancy-related liver disorders (OR = 2.34, 95% CI: 1.12–4.89, *p* < 0.05). These findings are illustrated in detail in Supplementary Table S5.

In women with explained RPL the following causes were detected: anatomic causes, 21.7%; endocrine causes, 15%; thrombophilias (hereditary and acquired), 6%; immunologic causes, 11.7%; parental chromosomal disorders, 1.8%; and environmental and health behaviors causes, 3.9%.

Finally, the pregnancy complications in women with RPL were stratified by the other main diagnostic categories, i.e., primary and secondary. In our study, 284 women had primary RPL and 147 women had secondary RPL. Women with secondary RPL had a higher rate of pregnancy complications (93/147, 63.26%) than women with primary RPL (138/284, 48.59%; OR = 1.822, 95% CI: 1.211–2.470, *p* < 0.005). However, no differences between women with primary and secondary RPL were found for any of the specific complications considered, with the exception of gestational diabetes mellitus that was more frequent in women with secondary RPL (OR = 0.40; 95% CI: 0.21–0.77, *p* < 0.01). These findings are shown in detail in Supplementary Table S6.

## 4. Discussion

The results of the present study show that women with RPL during their first gestation after the completion of the diagnostic workup had a significantly higher rate of several pregnancy complications than normal healthy women without RPL. Nearly all the pregnancy complications considered in our study occurred more frequently in women with RPL and that women with RPL had an increased risk to have multiple pregnancy complications than control women. To our knowledge, this aspect has been scarcely explored in women with RPL. These observations suggest that pregnancy in women with RPL could be considered high-risk in its entirety and support the general concept that these women could have a wide reproductive disorder not limited to early pregnancy establishment and maintenance; rather, it can be extended also to late gestation, once the implantation of the embryo and its initial development have been successfully established. However, this hypothesis could be in contrast with the high rate (86%) of live births observed in our study. There are several potential explanations for this. It is known that the final outcome of the successive pregnancy in women with RPL in terms of live births could be considered, all in all, satisfactory, particularly in those women with a limited number of previous losses [5,22,23]. This is the case of our study, in which the majority of women studied (332/431, 77.03%) had two or three previous losses, while the women with ≥4 previous losses, those at highest risk of an unfavorable outcome, were 99/431, (22.97%), i.e., less than one quarter of the overall population of women with RPL). It is also possible that the high rates of live births in our study women, albeit are within or near the high range reported in previous studies [4,5,22], are linked to complications of moderate severity; moreover, our live birth rates could be to some extent overestimated, since a part of our cohort of pregnant women (13.7% of women with RPL and 12.1% of control women) was lost at follow-up and this is a limitation of the present study.

The rate of spontaneous miscarriage in women with RPL was much higher than that found in control women. The risk of miscarriage increased by increasing the number of previous losses. The higher risk of fetal anomalies in women with RPL is in substantial accordance with the observations of previous studied carried out on this issue [24,25], even though other more recent studies could not find this association [8,26]. However, the postnatal genetic follow-up of the newborns was incomplete in our series and this limitation does not allows to draw firm conclusions on this issue. In our study, women with RPL had an increased risk of fetal growth restriction and intrauterine fetal demise compared to control women. These findings are in accordance, although to a variable extent, with many of the studied previously carried out [6,7,24,27]. However, several other studies could not demonstrate the above associations with RPL [26,28]. Similar considerations can be made for gestational diabetes mellitus, preeclampsia, placenta previa and abruptio placentae, conditions for which the association was either found to variable extent or not found at all [5,6,7,8,9,10,24,26,27,28,29,30]. Pregnancy-related liver disorders were found to be more frequent in women with RPL than in control women. This finding is in accordance with what has been observed by Cozzolino et al. [31].

In the present study there are additional major findings related to the specific population of women with RPL.

(1) By increasing the number of previous losses, the rates of women who became pregnant decreased, however the rates of women with pregnancy complications have the tendency to increase. Since the number of previous losses is considered an indicator of the severity of the RPL condition [3,32], it is possible that the biological factors underlying multiple pregnancy losses can continue to act by impairing the successive pregnancies, even though they have the strength to evolve towards advanced gestational ages. Further research is needed to check this hypothesis. A clear effect of the gravity of RPL condition, in terms of number of previous losses, has been shown in the case of selected pregnancy complications (Table 4).
(2) When the women with RPL were stratified according to the two major diagnostic categories, explained and unexplained, the rates of overall pregnancy complications were similar. However, the analysis carried out by specific complication revealed that the risk of preeclampsia and abruptio placentae was higher in women with unexplained RPL. A possible, plausible explanation for this finding—taken into account that the above conditions are linked since preeclampsia is a known major risk factor for abruptio placentae—is that in some or several women with unexplained RPL a disorder in the placentation could occur.
(3) When the women with RPL were stratified according to the other two major diagnostic categories, primary and secondary RPL, the rates of overall pregnancy complications were similar. However, the analysis carried out by specific complication revealed that the risk of GDM was higher in women with secondary than in women with primary RPL. A possible explanation for this finding could be that women with secondary RPL have been more exposed than women with primary RPL to the well-known diabetogenic effect of pregnancy that is exerted mainly in the second half of pregnancy, making them more susceptible for GDM in a successive pregnancy. This possibility is also supported by recent observation showing the association between high numbers of pregnancies and the increased prevalence of GDM [33].

On the basis of all the above consideration, it is clear that assessing the outcome of the first pregnancy after referral with the aim to establish a clear prognosis is highly problematic [3]. In fact, it is very difficult to make comparisons and fully explain the differences in the specific pregnancy complications observed between the studies, including the present one. This can be due to multiple reasons, including the heterogeneity of the studies with regard to the study design (retrospective/prospective), the inclusion/exclusion criteria, the different specific complications taken into account and their clear definitions, the stratification of women with RPL in specific subgroups, the potential impact of different therapeutic managements; in several studies a control group is lacking [4,5,27,29]. On the other hand, the major limitations of our study are the incomplete follow-up of the initially included women, particularly the pregnant ones, and the limited number of women with multiple pregnancy losses, i.e., ≥4. Another limitation of the present study is that the intrapartum complications of pregnancy, as well as the neonatal complications, have not been reported because they were not included in the design of the present study, whose aim was to gain and report as much information as possible on the prepartum outcome of the investigated subjects.

Finally, there is evidence suggesting that women with RPL are at increased risk of long-term cardiovascular complications [34], so that recently FIGO had published guidelines regarding long-term follow up on these women in order to decrease this risk [35]. We believe that this issue is worth to be investigated in depth.

## 5. Conclusions

The results of the present study show that women with RPL have an increased risk to develop pregnancy-related complications during the first gestation after their referral; their pregnancy should be considered at high-risk and deserves special attention and care, even though caution is needed before drawing firm conclusions on this relevant issue, as it has recently reported [34]. Clearly, further investigation is needed to fully clarify still many aspects of this important issue.

## Figures and Tables

**Table 1:** Definitions of pregnancy complications.

Complication	Definition
Threatened miscarriage	An abnormal vaginal bleeding and abdominal pain occurring before the 24 weeks in an otherwise ongoing pregnancy
Miscarriage	A spontaneous PL occurring before the 24 weeks gestation
Cervical insufficiency	CL < 25 mm by transvaginal ultrasound, or cervical changes detected on physical examination before 24 weeks of gestation [14]
Chromosomal abnormalities	Any detected alteration of the fetal karyotype and/or DNA
Fetal anomalies	Any structural/morphological abnormality detected by ultrasound
Oligohydramnios	AFV < 5% for GA, or AFI < 5 cm or MDP < 2 cm [15]
Polyhydramnios	A deepest vertical pocket of >8 cm or an AFI > 24 cm [16]
Fetal growth restriction	Fetus with an UEFW 5–10th percentile for GA, calculated using the IGC according to Snijders and Nicolaides [17]
Intrauterine fetal death	Fetal death at 24 weeks gestation or late
GDM	GDM was defined following WHO criteria [18]
Preeclampsia	Preeclampsia was defined according to ACOG 2013 [19]
Placenta previa/low-lying placenta	Defined according to the criteria of RCOG [20]
Placental abruptio	The premature separation of the placenta before delivery [21]
Pregnancy-related liver disorders	(a) HG; (b) ICP; (c) AFLP; (d) HELLP syndrome; (e) ALE; (f) BO
Preterm PROM	Premature rupture of the membranes before 37 weeks gestation

PL = pregnancy loss; CL = Cervical length; MDP = maximal deepest pocket; AFV = Amniotic fluid volume; AFI = Amniotic fluid index; MDP = maximum deepest pocket; GA = gestational age; UEFW = ultrasound estimated fetal weight; IGC = intrauterine growth curve; GDM = Gestational diabetes mellitus; WHO = World Health Organization; RCOG = Royal College of Obstetricians and Gynaecologists; ACOG = American of Obstetricians and Gynecologists; HG = hyperemesis gravidarum; ICP = intrahepatic cholestasis of pregnancy; AFLP = acute fatty liver of pregnancy; HELLP syndrome = hemolysis, elevated liver enzymes and low platelets count; ALE = isolated abnormal liver enzymes; BO = biliary obstruction by gallbladder stones; PROM = premature rupture of the membranes.

**Table 2:** General characteristics of study women.

	Group 1 Women with RPL in Pregnancy *(n)*	Group 2 (Controls) Healthy Pregnant Women *(n)*
Subjects	431	661
Age (years)	35.83 ± 5.95 *	31.70 ± 5.82
BMI (Kg/m^2^)	24.51 ± 4.66 *	23.41 ± 4.78
Cigarette smoking	56 (13%) **	126 (19%)
Ethnicity Caucasian	378 (87.7%)	551 (83.3%)
Asiatic	23 (5.4%)	47 (7.1%)
Hispanic	9 (2%)	24 (3.6%)
African	21 (4.9%)	39 (6%)
Number of pregnancy losses	1247	-
Miscarriages per women	2.89 ± 1.15	-
Women with 2 losses (%)	209 (48.5%)	-
Women with ≥3 losses (%)	222 (51.5%)	-
Women with RPL explained	259 (60.1%)	-
Women with RPL unexplained	172 (39.9%)	-
Women with primary RPL	284 (65.89%)	-
Women with secondary RPL	147 (34.11%)	-
Years of follow-up	2.58 ± 1.8°	2.51 ± 1.6

Data are expressed as means ± SD or percentages. * *p* < 0.001 vs. control (Student’s *t*-test); ** *p* < 0.05 vs. control (chi-square test). ° Not significant vs. control. BMI = Body mass index

**Table 3:** Pregnancy complication rates in women with RPL and control women. The factorial analysis of the effect size of age and BMI is reported as Δ% of the whole study population.

Type of Complication	Women with RPL (%)	Healthy Pregnant Women (C) (%)	OR (95%CI)	*p*-Value	Δ% by Age (RPL vs. C) *	Δ% by BMI (RPL vs. C) *
Threatened miscarriage	51 (11.8%)	26 (3.9%)	3.278 (2.01–5.34)	<0.0001	−0.9	1.9
Spontaneous miscarriage	55 (12.7%)	17 (2.5%)	5.541 (3.17–9.68)	<0.0001	4.8	3.3
Cervical insufficiency	21 (4.9%)	5 (0.75%)	6.72 (2.54–17.96)	<0.0001	−0.1	2.3
Chromosomal abnormalities	12 (2.8%)	3 (0.4%)	6.28 (1.76–22.39)	<0.005	1.4	3.3
Fetal anomalies	19 (4.24%)	12 (1.8%)	2.49 (1.19–5.19)	<0.01	−0.9	2.1
Oligohydramnios	13 (3%)	7 (1%)	2.90 (1.15–7.43)	<0.05	0.3	0.2
Polyhydramnios	4 (1%)	1 (0.15%)	6.18 (0.68–55.5)	NS	−0.8	1.5
Fetal growth restriction	14 (3.2%)	4 (0.6%)	5.51 (1.80–16.86)	<0.005	1.8	3.1
Intrauterine fetal death	5 (1.2%)	1 (0.15%)	7.74 (0.9–66.53)	<0.005	−0.1	0.9
Gestational diabetes mellitus	43 (10%)	29 (4.3%)	2.41 (1.48–3.93)	<0.0001	0.9	6.6
Preeclampsia	46(10.7%)	35 (5.3%)	2.13 (1.35–3.37)	<0.005	−2.5	8.9
Placenta previa	11 (2.5%)	5 (0.75%)	3.43 (1.18–9.95)	<0.05	0	1.5
Abruptio placentae	24 (5.6%)	19 (2.8%)	1.99 (1.07–3.69)	<0.05	0.5	0.4
Pregnancy-related liver disorders	32 (7.6%)	8 (1.2%)	6.54 (2.98–14.34)	<0.0001	1.5	1.6
Preterm PROM	28 (6.49%)	9 (1.36%)	51.4 (26.17–100.98)	<0.0001	0.9	3.6

* All the Δ% differences were significant with the exception of age in the case of placenta previa (Student’s *t* test). Positive values. indicate a size effect toward RPL women and negative values indicate a size effect toward control women.

**Table 4:** Risk of pregnancy complications in women with RPL stratified according to the number of previous losses. The ORs have been calculated for each group of women vs. controls.

Type of Complication	Women with 2 Losses OR (95% CI) *p*-Value	Women with 3 Losses OR (95% CI) *p*-Value	Women with >3 Losses OR (95% CI) *p*-Value
Threatened miscarriage	3.31 (1.87–5.88) *p* < 0.0001	2.39 (1.15–4.99) *p* < 0.02	4.36 (2.22–8.56) *p* < 0.0001
Spontaneous miscarriage	2.30 (1.08–4.91) *p* < 0.05	4.47 (2.11–9.47) *p* < 0.0005	16.47 (8.64–31.38) *p* < 0.0001
Cervical insufficiency	3.21 (0.92–11.21) *p* = 0.06, NS	7.91 (2.47–25.36) *p* < 0.001	13.12 (4.30–40.01) *p* < 0.0001
Chromosomal abnormalities	2.11 (0.35–12.76) *p* = 0.06, NS	9.29 (2.19–39.41) *p* < 0.005	11.07 (2.60–47.07) *p* < 0.002
Fetal anomalies	3.58 (1.61–7.98) *p* < 0.002	1.35 (0.37–4.86) *p* = 0.64, NS	1.69 (0.46–6.09) *p* = 0.42, NS
Oligohydramnios	3.23 (1.12–9.34) *p* < 0.05	2.33 (0.59–9.15) *p* = 0.22, NS	0.95 (0.11–7.93) *p* = 0.96, NS
Polyhydramnios	6.37 (0.57–70.68) *p* = 0.13, NS	5.40 (0.33–87.07) *p* = 0.23, NS	6.73 (0.41–108.55) *p* = 0.17, NS
Fetal growth restriction	4.85 (1.35–17.37) *p* < 0.02	4.10 (0.90–18.57) *p* = 0.06, NS	8.73 (2.30–33.11) *p* < 0.002
Intrauterine fetal death	9.61 (0.99–92.90) *p =* 0.05, NS	5.50 (0.34–88.53) *p* = 0.22, NS	6.73 (0.41–108.55) *p* = 0.17, NS
Gestational diabetes mellitus	2.39 (1.35–4.23) *p* < 0.005	2.14 (1.03–4.40) *p* < 0.05	2.17 (0.99–4.75) *p* = 0.05, NS
Preeclampsia	2.76 (1.63–4.67) *p* < 0.0002	1.93 (0.97–3.83) *p* = 0.05, NS	1.15 (0.47–2.21) *p* = 0.75, NS
Placenta previa	5.90 (1.95–17.81) *p* < 0.002	2.16 (0.41–11.30) *p* = 0.35, NS	0.59 (0.03–10.93) *p* = 0.73, NS
Abruptio placentae	1.17 (0.48–2.82) *p* = 0.72, NS	3.31 (1.53–7.16) *p* < 0.05	2.18 (0.84–5.59) *p* = 0.10, NS
Pregnancy-related liver disorders	4.97 (2.00–12.33) *p* < 0.001	9.64 (3.90–23.81) *p* < 0.0001	6.21 (2.20–17.52) *p* < 0.001
Preterm PROM	3.26 (1.27–8.32) *p* < 0.05	3.71 (1.29–10.63) *p* < 0.02	10.95 (4.54–26.37) *p* < 0.0001

NS = not significant.

**Table 5:** Number of concomitant pregnancy complications in study women.

	Women with RPL (*n* = 431) (%)	Healthy Pregnant Women (*n* = 661) (%)	OR (95% CI)	*p*-Value
Women with 1 complication	138 (32%)	101 (15.27%)	2.611 (1.948–3.500)	<0.0001
Women with 2 complications	54 (12.52%)	31 (4.61%)	2.910 (1.838–4.609)	<0.0001
Women with 3 complications	26 (6.03%)	6 (0.9%)	7.008 (2.859–17.174)	<0.0001
Women with 4 complications	11 (2.55%)	-	NA	
Women with 5 complications	2 (0.46%)	-	NA	

NA = not applicable.

## Supplementary Materials

The following are available online at https://www.mdpi.com/2077-0383/9/9/2833/s1, Supplementary Table S1: Detail of the factorial analysis of effect size of age on pregnancy complications in study women. Supplementary Table S2: Detail of the factorial analysis of effect size of BMI on pregnancy complications in study women. Supplementary Table S3: Detail of the occurrence of the pregnancy-related liver disorders. Supplementary Table S4: Rates of pregnancy complications in women with Recurrent Pregnancy Loss according to the number of previous pregnancy losses. Supplementary Table S5: Pregnancy complications in women with RPL according to the main diagnostic categories (Unexplained/Explained). Supplementary Table S6: Pregnancy complications in women with RPL according to the main diagnostic categories (Primary/Secondary).
