# Supplementary material for: Pregnancy-Related Complications in Women with Recurrent Pregnancy Loss: A Prospective Cohort Study
Source: J Clin Med. 2020 Sep 1;9(9):2833. doi: 10.3390/jcm9092833 (PMC7564138; doi:10.3390/jcm9092833)
Supplement: Supplementary file 1 [file jcm-09-02833-s001.pdf]

Supplementary Table S1: Detail of the factorial analysis of effect size of age on pregnancy complications in study women.

	AGE			
**Complication**	**Comparisons**	**Δ%**	**Student's t**	***p*-Value**
Threatened miscarriage	{Cases - Controls}	8.3	1084.449	< 0.0001
	{(Age > M) - (Age ≤ M)}	- 0.9	- 117.591	< 0.0001
	{(Cases - Controls) x ((Age >M) - (Age ≤M))}	2.7	352.773	< 0.0001
Miscarriage	{Cases - Controls}	11.1	1484.71	< 0.0001
	{(Age > M) - (Age ≤ M)}	4.8	642.037	< 0.0001
	{(Cases - Controls) x ((Age >M) - (Age ≤M))}	- 4.8	- 642.037	< 0.0001
Cervical insufficiency	{Cases - Controls}	4.2	923.141	< 0.0001
	{(Age > M) - (Age ≤ M)}	- 0.1	-21.98	< 0.0001
	{(Cases - Controls) x ((Age >M) - (Age ≤M))}	0.1	21.98	< 0.0001
Chromosomal abnormalities	{Cases - Controls}	2.5	700.609	< 0.0001
	{(Age > M) - (Age ≤ M)}	1.4	392.341	< 0.0001
	{(Cases - Controls) x ((Age >M) - (Age ≤M))}	2.5	700.609	< 0.0001
Fetal anomalies	{Cases - Controls}	3.7	695.604	< 0.0001
	{(Age > M) - (Age ≤ M)}	- 0.9	-169.201	< 0.0001
	{(Cases - Controls) x ((Age >M) - (Age ≤M))}	1.6	300.802	< 0.0001
Oligohydramnios	{Cases - Controls}	2.1	508.698	< 0.0001
	{(Age > M) - (Age ≤ M)}	0.3	72.671	< 0.0001
	{(Cases - Controls) x ((Age >M) - (Age ≤M))}	2.4	581.369	< 0.0001
Polyhydramnios	{Cases - Controls}	0.2	71.741	< 0.0001
	{(Age > M) - (Age ≤ M)}	- 0.8	- 286.966	< 0.0001
	{(Cases - Controls) x ((Age >M) - (Age ≤M))}	1.5	538.061	< 0.0001
Fetal growth restriction	{Cases - Controls}	0.1	19.632	< 0.0001
	{(Age > M) - (Age ≤ M)}	1.8	353.384	< 0.0001
	{(Cases - Controls) x ((Age >M) - (Age ≤M))}	-1.8	-353.384	< 0.0001
Intrauterine fetal death	{Cases - Controls}	1.0	439.239	< 0.0001
	{(Age > M) - (Age ≤ M)}	- 0.1	- 43.924	< 0.0001
	{(Cases - Controls) x ((Age >M) - (Age ≤M))}	0.6	263.543	< 0.0001
GDM	{Cases - Controls}	6.3	830.45	< 0.0001
	{(Age > M) - (Age ≤ M)}	0.9	118.636	< 0.0001
	{(Cases - Controls) x((Age >M) - (Age ≤M))}	3.2	421.816	< 0.0001
Preeclampsia	{Cases - Controls}	5.6	710.734	< 0.0001
	{(Age > M) - (Age ≤ M)}	- 2.5	-317.292	< 0.0001
	{(Cases - Controls) x ((Age >M) - (Age ≤M))}	2.5	317.292	< 0.0001
Placentaprevia/ Low-lying placenta	{Cases - Controls}	1.9	514.398	< 0.0001
	{(Age > M) - (Age ≤ M)}	0	0	n.s.
	{(Cases - Controls) x ((Age >M) - (Age ≤M))}	0.4	108.294	< 0.0001
Placental abruptio	{Cases - Controls}	2.3	397.204	< 0.0001
	{(Age > M) - (Age ≤ M)}	0.5	86.349	< 0.0001
	{(Cases - Controls) x ((Age >M) - (Age ≤M))}	2.9	500.823	< 0.0001
Pregnancy-related liver disorders	{Cases - Controls}	6.5	1150.595	< 0.0001
	{(Age > M) - (Age ≤ M)}	1.5	265.522	< 0.0001
	{(Cases - Controls) x ((Age >M) - (Age ≤M))}	3.4	601.85	< 0.0001
Preterm Prom	{Cases - Controls}	5.1	947.299	< 0.0001
	{(Age > M) - (Age ≤ M)}	0.9	167.17	< 0.0001
	{(Cases - Controls) x ((Age >M) - (Age ≤M))}	4.5	835.852	< 0.0001

M = Median; For each specific issue in the first line it has been calculated the increased rate of complication occurrence between cases and controls; in the second line it has been calculated the effect of age by stratifying the ages of cases and controls according to the median of the whole population; in the third line it has been calculated the interaction between the rates reported in the two preceding lines. n.s. = not significant.

Supplementary Table S2: Detail of the factorial analysis of effect size of BMI on pregnancy complications in study women.

	BMI			
**Complication**	**Comparison**	**Δ%**	**Student's t**	***p*-value**
Threatened miscarriage	{Cases - Controls}	12.1	784.581	< 0.0001
	{(BMI > M) - (BMI ≤ M)}	1.9	123.199	< 0.0001
	{(Cases - Controls) x ((BMI >M) - (BMI ≤M))}	1.1	71.326	< 0.0001
Miscarriage	{Cases - Controls}	18.4	1153.341	< 0.0001
	{(BMI> M) - (BMI ≤ M)}	3.3	346.167	< 0.0001
	{(Cases - Controls) x ((BMI >M) - (BMI ≤M))}	-1.7	-178.328	< 0.0001
Cervical insufficiency	{Cases - Controls}	4.6	423.087	< 0.0001
	{(BMI > M) - (BMI ≤ M)}	2.3	211.543	< 0.0001
	{(Cases - Controls) x ((BMI >M) - (BMI ≤M))}	0.3	27.593	< 0.0001
Chromosomal abnormalities	{Cases - Controls}	11.8	1003.351	< 0.0001
	{(BMI > M) - (BMI ≤ M)}	3.3	280.598	< 0.0001
	{(Cases - Controls) x ((BMI >M) - (BMI ≤M))}	4.8	408.143	< 0.0001
Fetal anomalies	{Cases - Controls}	3.0	269.727	< 0.0001
	{(BMI > M) - (BMI ≤ M)}	2.1	188.809	< 0.0001
	{(Cases - Controls) x ((BMI >M) - (BMI ≤M))}	1.9	170.827	< 0.0001
OLIGOAMN	{Cases - Controls}	2.5	314.231	< 0.0001
	{(BMI > M) - (BMI ≤ M)}	0.2	25.139	< 0.0001
	{(Cases - Controls) x ((BMI >M) - (BMI ≤M))}	-0.3	-37.708	< 0.0001
Polyhydramnios	{Cases - Controls}	1.1	238.692	< 0.0001
	{(BMI > Median) - (BMI ≤ Median)}	1.5	325.489	< 0.0001
	{(Cases - Controls) x ((BMI >M) - (BMI ≤M))}	0.3	65.098	< 0.0001
Fetal growth restriction	{Cases - Controls}	3.8	431.183	< 0.0001
	{(BMI > M) - (BMI ≤ M)}	3.1	351.755	< 0.0001
	{(Cases - Controls) x ((BMI >M) - (BMI ≤M))}	-0.3	-34.041	< 0.0001
Intrauterine fetal death	{Cases - Controls}	1.1	238.57	< 0.0001
	{(BMI > M) - (BMI ≤ M)}	0.9	195.194	< 0.0001
	{(Cases - Controls) x ((BMI >M) - (BMI ≤M))}	0.9	195.194	< 0.0001
GDM	{Cases - Controls}	8.7	554.605	< 0.0001
	{(BMI > M) - (BMI ≤ M)}	6.6	420.735	< 0.0001
	{(Cases - Controls) x ((BMI >M) - (BMI ≤M))}	0.7	44.623	< 0.0001
Preeclampsia	{Cases - Controls}	9.9	603.84	< 0.0001
	{(BMI > M) - (BMI ≤ M)}	8.9	542.846	< 0.0001
	{(Cases - Controls) x ((BMI >M) - (BMI ≤M))}	0.8	48.795	< 0.0001
Placenta previa/ Low-lying placenta	{Cases - Controls}	3.4	397.276	< 0.0001
	{(BMI > M) - (BMI ≤ M)}	1.5	175.269	< 0.0001
	{(Cases - Controls) x ((BMI >M) - (BMI ≤M))}	-0.5	-58.423	< 0.0001
Placental abruptio	{Cases - Controls}	4.7	373.649	< 0.0001
	{(BMI > M) - (BMI ≤ M)}	0.4	31.8	< 0.0001
	{(Cases - Controls) x ((BMI >M) - (BMI ≤M))}	0.3	23.85	< 0.0001
Pregnancy-related liver disorders	{Cases - Controls}	7.8	683.282	< 0.0001
	{(BMI > M) - (BMI ≤ M)}	1.6	140.16	< 0.0001
	{(Cases - Controls) x ((BMI >M) - (BMI ≤M))}	-1.5	-131.4	< 0.0001
Preterm Prom	{Cases - Controls}	4.6	423.087	< 0.0001
	{(BMI > M) - (BMI ≤ M)}	3.6	331.111	< 0.0001
	{(Cases - Controls) x ((BMI >M) - (BMI ≤M))}	0.3	27.593	< 0.0001

M = Median; For each specific issue in the first line it has been calculated the increased rate of complication occurrence between cases and controls; in the second line it has been calculated the effect of age by stratifying the ages of cases and controls according to the median of the whole population; in the third line it has been calculated the interaction between the rates reported in the two preceding lines. n.s. = not significant.

Supplementary Table S3: Detail of the occurrence of the pregnancy-related liver disorders.

Pregnancy-related liver disorder	Number of cases
Intrahepatic cholestasis of pregnancy	8 cases
Acute fatty liver of pregnancy	1 case
Isolated abnormal liver enzymes	29 cases
Biliary obstruction by gallbladder stones	2 cases

The above list include the overall findings observed in all the study women and are not limited to women with RPL. They have been stratified as a whole and not by specific complication between women of the two study groups due to the very low numbers of events in the case of AFLP and BO.

Supplementary Table S4: Rates of pregnancy complications in women with RPL according to the number of previous pregnancy losses.

	**Number of Previous Losses (%)**							
	**2 Losses**	**3 Losses**	**4 Losses**	**5 Losses**	**6 Losses**	**7 Losses**	8 Losses	9 Losses
Women who become pregnant (n = 431)	209 (48.4 %)	123 (28.5 %)	60 (13.9 %)	25 (5.8 %)	7 (1.6 %)	4 (0.9 %)	2 (0.4%)	1 (0.2%)
Women with pregnancy complications (n = 231)	99 (47.3%)	68 (55.3%)	39 (65%)	15 (60%)	5 (71.4%)	4 (100%)	1 (50%)	N.A.

N.A. = not available; r = 0.993, $p < 0.0001$.

Supplementary Table S5 – Pregnancy complications in women with RPL according to the main diagnostic categories - Unexplained /Explained.

	**Women with unexplained RPL [n = 172] (%)**	**Women with explained RPL [n = 259] (%)**	**OR (95% CI)**	***p-value***
Women with complications	94 (54.65%)	137 (52.89%)		
Threatened miscarriage	21 (12.2%)	30 (11.6%)	1.06 (0.58–1.92)	0.84, NS
Spontaneous miscarriage	20 (11.6%)	35 (13.5%)	0.84 (0.46–1.51)	0.56, NS
Cervical insufficiency	11 (6.4%)	10 (3.8%)	1.70 (0.70–4.09)	0.23, NS
Chromosomal/genetic abnormalities	2 (1.2%)	10 (3.8%)	0.29 (0.06–1.35)	0.11, NS
Fetal anomalies	7 (4.0%)	12 (4.6%)	0.87 (0.33–2.26)	0.78, NS
Oligohydramnios	5 (2.9%)	8 (3.1%)	0.93 (0.30–2.92)	0.91, NS
Polyhydramnios	2 (1.2%)	2 (0.8%)	1.51 (0.21–10.83)	0.68, NS
Fetal growth restriction	6 (3.5%)	8 (3.1%)	1.13 (0.38–3.32)	0.81, NS
Intrauterine fetal death	4 (2.3%)	1 (3.4%)	6.14 (0.68-55.43)	0.10, NS
Gestational diabetes mellitus	15 (8.7%)	28 (10.8%)	0.78 (0.40–1.52)	0.47, NS
Preeclampsia	27 (15.7%)	19 (7.4%)	2.35 (1.26–4.38)	< 0.05
Placenta previa	5 (2.9%)	6 (2.3%)	1.26 (0.37–4.20)	0.70, NS
Abruptio placentae	8 (4.6%)	16 (6.2%)	0.74 (0.30–1.77)	0.49, NS
Pregnancy-related liver disorders	19 (11.0%)	13 (5.0%)	2.34 (1.12–4.89)	< 0.05
Preterm PROM	11 (6.3%)	17 (6.5%)	0.97 (0.44–2.13)	0.94, NS

NS = Not significant.

Supplementary Table S6: Pregnancy complications in women with RPL according to the main diagnostic categories – Primary/Secondary.

	Women with primary RPL [n = 284] (%)	Women with secondary RPL [n = 147] (%)	OR (95% CI)	*p-value*
**Women with complications**	138 (48.59%)	93 (63.26%)		
Threatened miscarriage	31 (10.91%)	20 (13.6%)	0.77 (0.42–1.41)	0.41, NS
Spontaneous miscarriage	35 (12.32%)	20 (13.6%)	0.89 (0.49–1.60)	0.70, NS
Cervical insufficiency	12 (4.22%)	9 (6.11%)	0.69 (0.28–1.69)	0.42, NS
Chromosomal/genetic abnormalities	6 (2.11%)	6 (4.08%)	0.50 [0.16–1.60]	0.24, NS
Fetal anomalies	11 (3.87%)	8 (5.44%)	0.70 (0.27–1.78)	0.45, NS
Oligohydramnios	6 (2.11%)	7 (4.76%)	0.43 (0.14–1.30)	0.13, NS
Polyhydramnios	1 (0.35%)	3 (2.04%)	0.16 (0.01–1.64)	0.12, NS
Fetal growth restriction	9 (3.16%)	5 (3.40%)	0.92 (0.30–2.82)	0.89, NS
Intrauterine fetal death	4 (1.4%)	1 (0.68%)	2.08 (0.23–18.83)	0.51, NS
Gestational diabetes mellitus	20 (7.04%)	23 (15.64%)	0.40 (0.21–0.77)	< 0.01
Preclampsia	29 (10.21%)	17 (11.56%)	0.86 (0.46–1.64)	0.66, NS
Placenta previa	6 (2.11%)	5 (3.40%)	0.61 (0.18–2.04)	0.42, NS
Abruptio placentae	12 (4.22%)	12 (8.16%)	0.49 (0.21–1.13)	0.09, NS
Pregnancy-related liver disorders	21 (7.39%)	11 (7.48%)	0.98 (0.46–2.10)	0.97, NS
Preterm PROM	21 (7.39%)	7 (4.76%)	1.59 (0.66–3.84)	0.29, NS

NS = not significant.
